# Experimental African Trypanosome Infection by Needle Passage or Natural Tsetse Fly Challenge Thwarts the Development of Collagen-Induced Arthritis in DBA/1 Prone Mice via an Impairment of Antigen Specific B Cell Autoantibody Titers

**DOI:** 10.1371/journal.pone.0130431

**Published:** 2015-06-25

**Authors:** Carl De Trez, Brunette Katsandegwaza, Guy Caljon, Stefan Magez

**Affiliations:** 1 Research Unit of Cellular and Molecular Immunology, Vrije Universiteit Brussel, Brussels, Belgium; 2 VIB Department of Structural Biology, Brussels, Belgium; 3 Unit of Veterinary Protozoology, Department of Biomedical Sciences, Institute of Tropical Medicine Antwerp (ITM), Antwerp, Belgium; 4 Unit of Cellular and Molecular Immunology, Vrije Universiteit Brussel (VUB), Brussels, Belgium; 5 Laboratory of Myeloid Cell Immunology, Vlaams Instituut voor Biotechnologie (VIB), Brussels, Belgium; Wayne State University, UNITED STATES

## Abstract

Collagen-induced arthritis is a B cell-mediated autoimmune disease. Recently published studies have demonstrated that in some rare cases pathogens can confer protection from autoimmunity. *Trypanosoma brucei* parasites are tsetse fly transmitted extracellular protozoans causing sleeping sickness disease in humans and Nagana in livestock in sub-Saharan endemic areas. In the past, we demonstrated that trypanosome infections impair B cell homeostasis and abolish vaccine-induced protection against unrelated antigens. Hence, here we hypothesized that trypanosome infection can affect the onset of CIA by specifically dampening specific B-cell responses and type II collagen antibody titers in DBA/1 prone mice. We observed a substantial delay in the onset of collagen-induced arthritis in *T*. *brucei*-infected DBA/1 mice that correlates with a drastic decrease of type II collagen titers of the different IgG isotypes in the serum. Treatment of infected mice with Berenil, a trypanocidal drug, restored the development of CIA-associated clinical symptoms. Interestingly, these data were confirmed by the challenge of immunized DBA/1 prone mice with *T*. *brucei*-infected tsetse flies. Together, these results demonstrate that *T*. *brucei* infection is impairing the maintenance of the antigen specific plasma B cell pool driving the development of CIA in DBA/1 prone mice.

## Introduction

Recently, epidemiologists have observed a low occurrence of infectious diseases, coinciding with an increase prevalence of autoimmune diseases in the developed world, whereas they found the opposite, namely high incidence of infections associated to low rate of autoimmunity, in the developing countries. The reason is due to the fact that the developed world has managed to eradicate most infectious diseases, but has concomitantly witnessed a rise in autoimmune diseases, while the developing countries have still to battle with a number of infectious diseases with a very small percentage of autoimmune diseases [1]. These observations have led to the hygiene hypothesis, which states that the absence of early childhood exposure to infectious pathogens may give rise to an increased susceptibility to the natural development of autoimmune diseases and allergy [2]. In other words, infectious agents are constantly reshaping the immune system via the modulation of its different protagonists as well as the way they act.

Rheumatoid Arthritis (RA) is an auto-immune disease characterized by a systemic chronic inflammation, which primarily affects the joints [3]. Although the exact mechanisms implicated in RA are still unclear, numerous immune cell types, e.g. B cells, T cells, macrophages have been involved in its pathogenesis [4] [5]. More specifically, the presence of autoreactive B cells to Type II Collagen (CII), rheumatoid factor and anti-cyclic citrullinated peptide in the sera of RA patients is associated with a higher risk of mortality and morbidity as well as more severe articular cartilage disease [6].

Collagen-induced arthritis (CIA) is one of the most widely used animal models to study RA in humans. B cells play a major role in the initiation of CIA as B cell-deficient mice do not develop CIA while anti-CII T cell responses are preserved [7] [8]. At the same time, the presence of alphabeta T cells is necessary for the induction of CIA and the IgG responses towards CII [9]. CIA is inducible in DBA/1 prone mice through immunization with heterologous CII emulsified in adjuvant and major clinical symptoms are paw swelling, cartilage damage and bone erosion [10]. The major role of B cells is the production of arthritogenic anti-CII specific antibodies (Abs) of different isotypes, mainly IgG2a and IgG2c, that can bind to cartilage and induce arthritis [11].

Parasitic infections are typically associated with a modulation of the host antibody response, e.g. polyclonal B cell activation, modulation of B cell lymphopoiesis [12]. *Trypanosoma brucei* belongs to the family of African trypanosomes (AT), which are vector-borne extracellular protozoan parasites to humans and livestock and are transmitted by tsetse flies [13]. *T*. *brucei* infection in humans is the causative agent of sleeping sickness disease [13]. Trypanosomes also infect cattle, and have a huge economic impact with a loss of over US $2 billion per year in Africa alone, making it a parasite of major concern especially in rural Africa [13]. *T*. *brucei* parasites have evolved numerous immune evasion mechanisms in order to establish chronic infection within its host. Using an *in vivo* mouse model of infection, our laboratory has also demonstrated that *T*. *brucei* infection causes the ablation of B cell lymphopoiesis in primary and secondary lymphoid organs as well as the loss of memory recall response against unrelated antigens [14]. To this end, we tested this hygiene hypothesis by evaluating if a Trypanosome infection affects the onset of CIA by specifically impacting specific CII autoantibody titers.

## Material and Methods

### Ethics statement

All experiments complied with the ECPVA guidelines (CETS n° 123) and were approved by the VUB Ethical Committee (Permit Number: 10-220-13). Breeding and experimental work with tsetse flies was approved by the Scientific Institute Public Health department Biosafety and Biotechnology (SBB 219.2007/1410). To minimize mouse suffering and distress during blood sampling, all animals were anaesthetized with isoflurane using a UNO—Univentor Anaesthesia Unit according to the manufacturer`s protocol. Mice were monitored on a daily basis and no unexpected deaths were observed. Humane endpoints were used during the study, based on weight loss—animals with >25% weight loss were sacrificed using carbon dioxide treatment.

### Mice and immunization

Collagen-induced arthritis model: male DBA/1 mice (Janvier, France) were immunized with 100μg of type II collagen (CII) (Sigma) emulsified in Complete Freund’s Adjuvant (CFA) intradermally into the tail. Three weeks post-induction, immunized mice were boosted with 100μg of CII in Incomplete Freund’s Adjuvant (IFA). The mice were monitored and evaluated two to three times per week for arthritic incidences. Scoring was done based on the severity and the number of limbs exhibiting arthritic symptoms and was assigned as follows: 0 = No evidence of erythema and swelling, 1 = Erythema and mild swelling confined to the tarsals or ankle joint, 2 = Erythema and mild swelling extending from the ankle to the tarsals, 3 = Erythema and moderate swelling extending from the ankle to metatarsal joints, 4 = Erythema and severe swelling encompass the ankle, foot and digits, or ankylosis of the limb. The mice were maintained under specific pathogen-free conditions with an air conditioning system and a 12-h light/12-h dark cycle. The mice had free access to food and water during the experiments.

### Parasites, infection and treatment

Clonal pleomorphic *T*. *brucei* AnTat 1.1E parasites were a kind gift from N. Van Meirvenne (Institute for Tropical Medicine, Belgium) and stored at −80°C. Tsetse flies infected with non-clonal *T*. *brucei* AnTAR1 parasites were maintained at the Institute of Tropical Medicine. DBA/1 male mice (8 to 10 weeks old) were infected with 5000 AnTat1.1E trypanosomes intraperitonealy (i.p.) or using one individual tsetse fly with a mature salivary gland infection which was allowed to feed per mouse fourteen days post-priming with CII emulsified in CFA (day 0). Twenty-eight and 35 to 38 days post-CII in CFA priming, which correspond to 14 days post-tsetse fly and 21 to 24 days post-i.p. infection, respectively, both infected and uninfected mice were treated i.p. with diminazene aceturate (Berenil, 40 mg/kg, Sigma Aldrich, St. Louis, MO, USA) in PBS. Parasites were counted via blood dilution (1/200 in PBS) every 2 to 4 days using a counting chamber and a light microscope. Blood was drawn via the tail at different time-points for serological analyses.

### Enzyme-linked immunosorbant assay (ELISA) technique

96 well MicroWell MaxiSorp flat bottom plates (Sigma-Aldrich) were coated with 2μg/ml of antigen (CII, Sigma-Aldrich)) and incubated overnight at 4°C. The following day the plates were washed and blocked. Serial serum dilutions were added to the plates and incubated overnight at 4°C. The next day, plates were incubated with anti-mouse IgG1, IgG2a, IgG2b, IgG3 and IgM isotypes coupled to Horse Radish peroxydase (Lo-Imex, Louvain, BE), revealed using a TMB kit (BD Biosciences) and read at 450nm using a ELISA plate reader [15, 16].

### Statistics

The GraphPad Prism 4.0 software was used for statistical analyses (Two-way ANOVA or student *t*-test). Values are expressed as mean ± SEM. Values of p≤0.05 are considered statistically significant, where * = p≤0.05, ** = p≤0.01 and *** = p≤0.001.

## Results

### Trypanosomosis drastically delays the ongoing development of CIA

**Fig 1:**
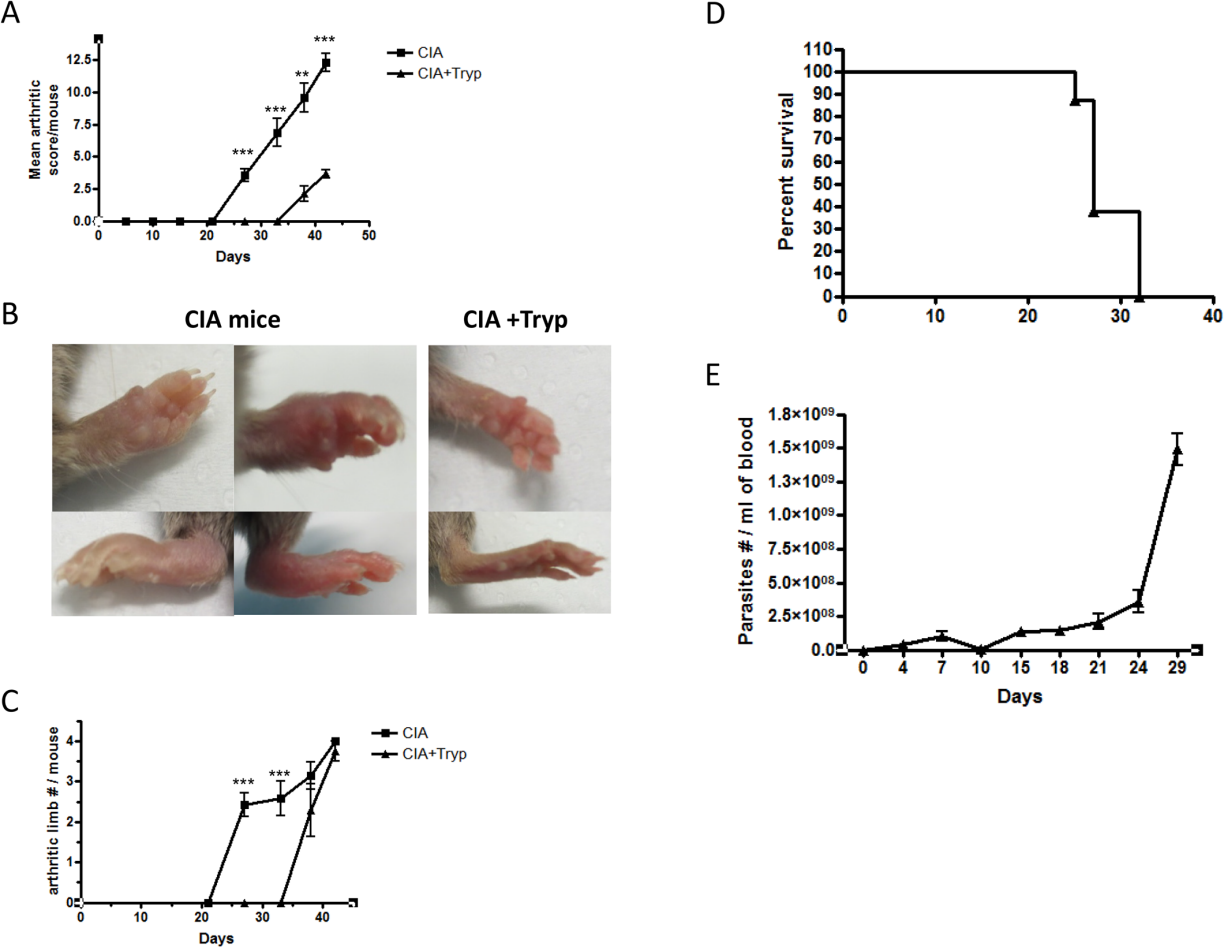
Trypanosomosis substantially delays the onset of CIA in DBA/1 prone mice. DBA/1 mice were immunized primarily with CII in CFA on day 0 and secondarily with CII in IFA on day 21. One group of mice was infected with *T*. *brucei* (filled triangle) on day 14 i.p. and the other was left untreated (filled square). (**A**) Average clinical score and (**C**) the number of arthritic limb per mouse were followed starting at day 0. (**B**) Representative pictures of arthritic limbs at day 42. Graphs show the mean ± SD from at least n = 6 mice per group and the data are representative of three independent experiments. (D&E) DBA/1 mice were infected with *T*. *brucei* i.p. and (**D**) survival and (**E**) blood parasite counts were monitored. Graphs show the mean ± SD from at least n = 6 mice per group and the data are representative of two independent experiments.

As our group already reported that *T*. *brucei* infection alters B cell homeostasis, we investigated if trypanosomosis was also able to modulate the course of B cell-mediated CIA in DBA/1 prone mice [14, 17]. In order to avoid any interference between the parasite and the priming of the anti-CII immune response, mice were infected with *T*. *brucei* two weeks post-immunization with CII emulsified in CFA. The results demonstrated a significant delay in CIA development in the infected group, which is characterized by a significant lower mean of arthritic lesion score at day 42 (Fig 1A). Inflamed limb pictures taken at the same time points clearly confirmed the protective effect of *T*. *brucei* infection on the development of CIA (Fig 1B). A delay in the incidence of arthritis defined by the number of arthritic limbs per mouse is also observed (Fig 1C). Trypanosome infected DBA/1 prone mice started to succumb from uncontrolled parasitemia from 25 days post-infection (approximately 40 days post-immunization) onwards (Fig 1D and 1E). Therefore we could not investigate CIA development in this particular group at a later time point. Together, these results demonstrate that trypanosomosis significantly delays the development of CIA in DBA/1 prone mice.

### *T*. *brucei* infection reduces the titers of circulating anti-Type II collagen IgGs

**Fig 2:**
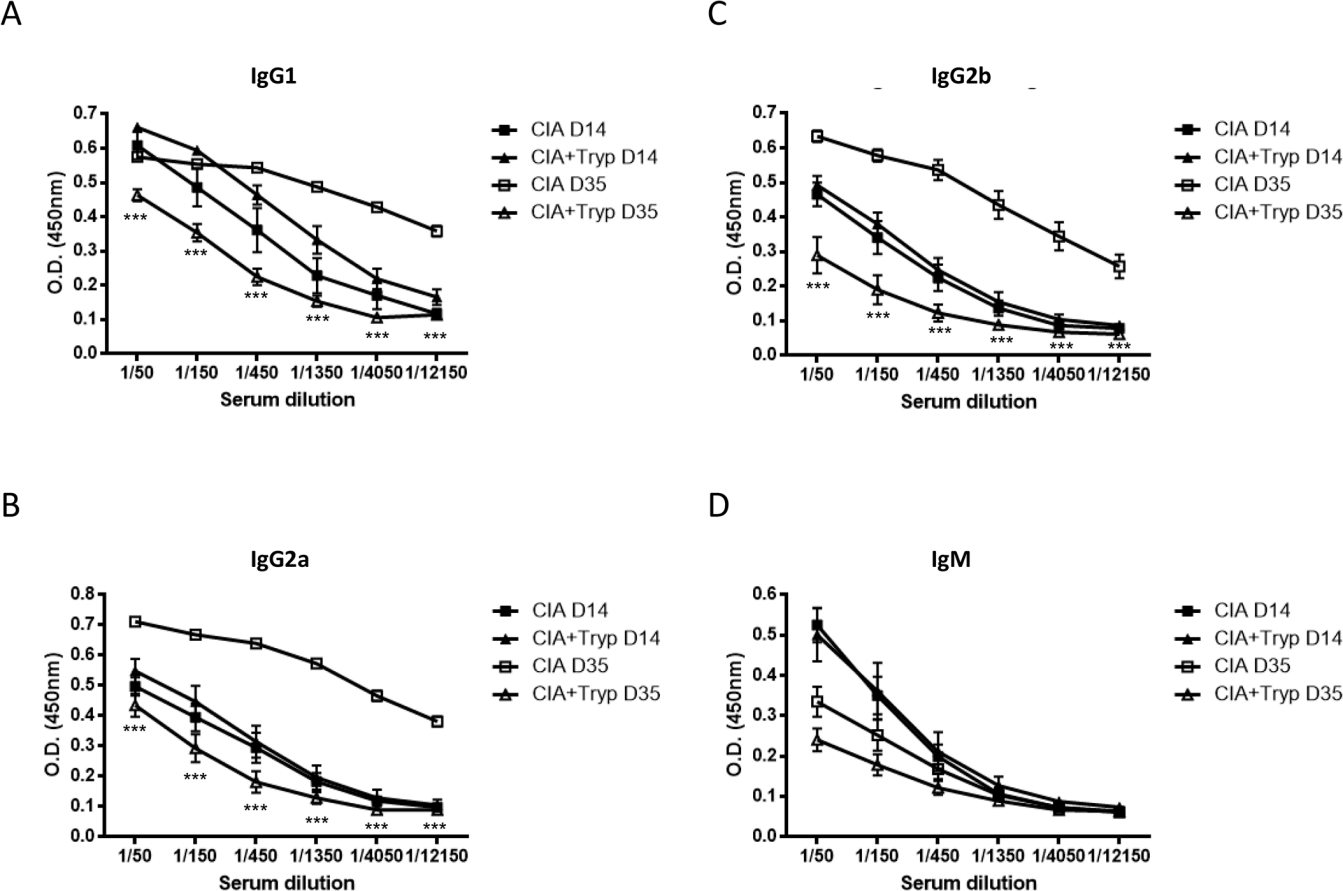
Trypanosomosis drastically affects the titers of circulating anti-CII IgG Abs. DBA/1 mice were immunized primarily with CII in CFA on day 0 and secondarily with CII in IFA on day 21. One group of mice was infected with *T*. *brucei* (filled and open triangle) on day 14 i.p. and the other was left untreated (filled and open square). Serial serum dilution levels of anti-CII (**A**) IgG1, (**B**) IgG2a, (**C**) IgG2b and (**D**) IgM Abs were measured by ELISA on day 14 (before infection, filled square and triangle) and day 35 (open square and triangle). Graphs show the mean ± SD from at least n = 6 mice per group and the data are representative of two independent experiments.

As mentioned previously, CIA is a B cell dependent autoimmune disease as B cell deficient mice do not develop type II collagen-induced arthritis [7]. As *T*. *brucei* infection has been shown to impact B cell homeostasis in other mouse strains, we decided to follow the levels of circulating specific anti-CII Abs of various isotypes before infection, namely day 14 post CII/CFA immunization, and before the mice start dying from the infection, round day 35 post-immunization (day 20 post-infection). As expected, before *T*. *brucei* infection, the titers of specific anti-CII IgM, IgG1, IgG2a and IgG2b Ab level are similar in both CIA-induced groups (Fig 2A, 2B and 2C). However, 35 days post-CIA in CFA priming (day 21 post-trypanosome infection), we observed a significant decrease (between 80- and 240-fold) in all anti-CII IgG isotypes titers between the infected group compared to the uninfected group (Fig 2A, 2B and 2C), but not for anti-CII specific IgM levels (Fig 2D). Hence, specific IgM level was the only isotype that seems to decrease during CIA development, which is in contrast to IgG1, IgG2a and IgG2b specific Ab titers. Collectively, the data suggested that the reduced onset of CIA in *T*. *brucei*-infected mice correlates with decreased levels of circulating anti-CII specific antibodies.

### Treatment of *T*. *brucei*-infected mice with the anti-trypanosomal drug, Berenil, restores the development of CIA

**Fig 3:**
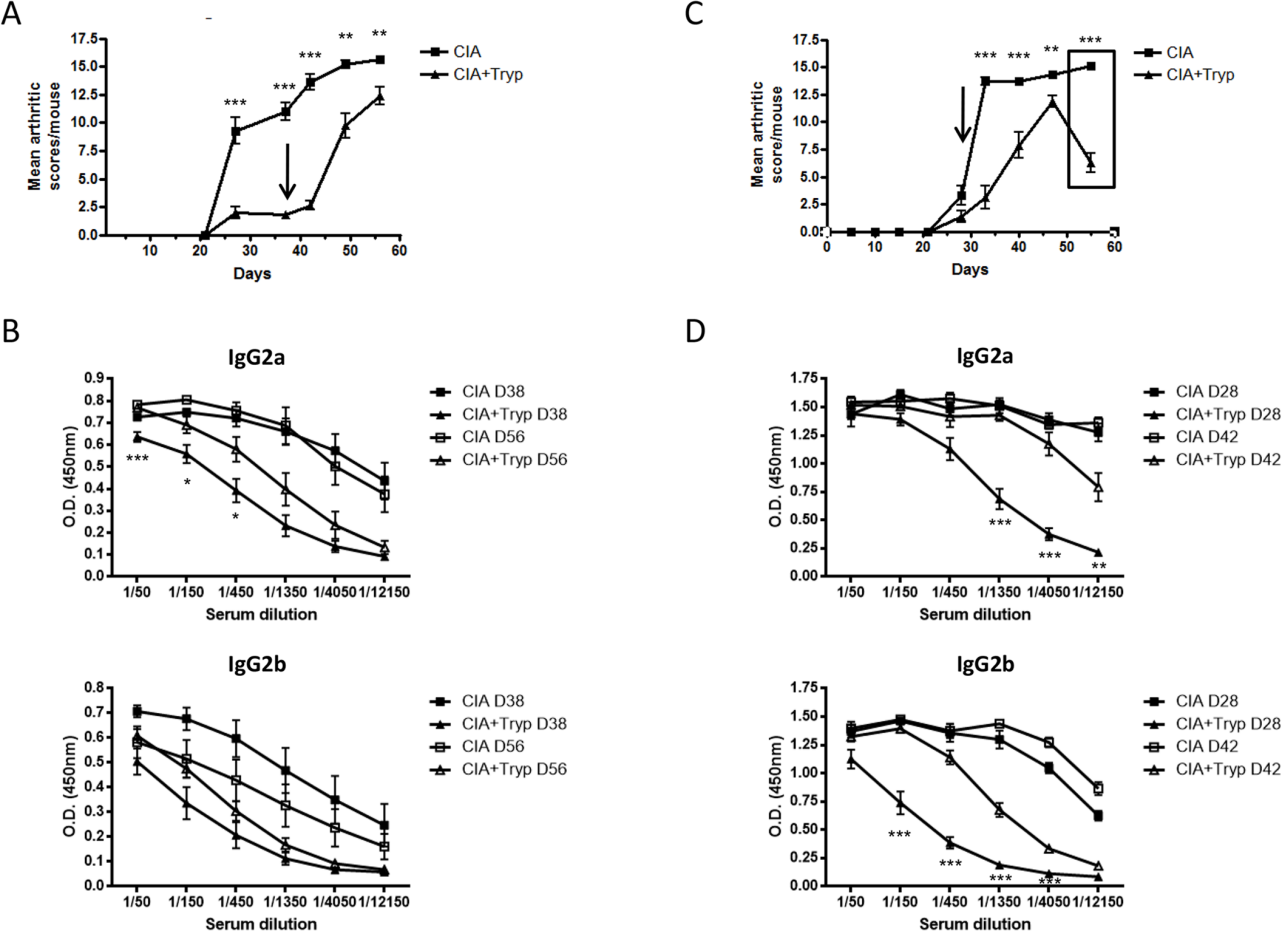
Berenil treatment restores the appearance of the CIA-associated clinical symptoms. DBA/1 mice were immunized primarily with CII in CFA on day 0 and secondarily with CII in IFA on day 21. One group of mice out of two was infected with *T*. *brucei* (filled and open triangle) on day 14 either i.p. or using tsetse flies and the other was left untreated (filled and open square). Both groups were treated with the trypanocidal drug, Berenil, at (**A**,**B**) day 38 (i.p.) or (**C,D**) day 28 (tsetse). (**A**) The average clinical per mouse score was followed from day 0. (**B**) Serial serum dilution levels of anti-CII IgG2a and IgG2b Abs were measured by ELISA on day 38 (before Berenil, filled square and triangle) and day 56 (open square and triangle). (**C**) Clinical score was followed from day 0 and (**D**) serial serum dilution levels of anti-CII IgG2a and IgG2b Abs were measured by ELISA on day 28 (before Berenil, filled square and triangle) and day 42 (open square and triangle). Arrows represent the day when trypanocidal drug Berenil was administered. Graphs show the mean ± SD from at least n = 6 mice per group and the data are representative of two independent experiments.

As all *T*. *brucei-*infected DBA/1 mice die within approximately 45 days post-CIA induction (30 days post-infection), we decided to treat infected mice with the anti-trypanosomal drug, Berenil, before the onset of death round day 35 post-immunization (day 21 post-infection, see arrow) and assess the development of CIA. It is important to note that the uninfected group also received the Berenil treatment in order to avoid any unspecific effects of this drug. Interestingly, *T*. *brucei* clearance from the blood circulation induces the restoration of CIA clinical symptoms. Indeed, starting one week after Berenil treatment, a gradual but drastic enhancement of the mean arthritic score per mouse was observed in the group of mice that were infected (Fig 3A). In agreement with these results, a significant increase in the titers of anti-CII Abs belonging to the IgG2a isotype, one the main isotypes implicated in CIA development [11], was observed between day 38 and 56 post-CIA induction in the CIA-primed and infected group of mice (Fig 3B).

The usual route of infection in the *T*. *brucei* mouse model is the intraperitoneal (i.p.) administration. In order to avoid any biases and to strengthen the physiological impact of our results, we decided to monitor the outcome of CIA in mice following infection using *T*. *brucei*-infected tsetse flies. It is important to note that the Berenil treatment was administered two weeks post-infection instead of three as the fly infection is more virulent (personal observation). Like the i.p. infection, the natural route of infection is also able to dampen the development of CIA (Fig 3C), as characterized by a slower development of CIA in these mice. The more rapid increase of the CIA score following the natural route of infection might be due to the fact that Berenil treatment was administered two weeks post-infection (day 28 post-immunization, see arrow) instead of three. By day 47 post-CII priming, the mean arthritic score per mouse of the infected group tended to catch up with the one of the uninfected group as the average of both scores are well above 10. As shown previously, we observed that a physiological infection also mediates a drastic decrease in anti-CII specific IgG2a and IgG2b antibodies (Fig 3D) by day 28 post-immunization (14 days post-infection), which coincided with a delay in the onset of CIA. Following tsetse fly infection, the drug treatment restored anti-CII specific IgG2a and, to some extent, IgG2b antibody titers, which correlates with the drastic increase of the mean arthritic score per mouse.

Surprisingly, 47 days post-CIA induction, we observed a decline in the mean arthritic score per mouse in mice that were “physiologically” infected and treated with Berenil (box Fig 3C). The most obvious explanation was that the drug could not sterilize the host from the parasite. Indeed, we demonstrated a relapse of *T*. *brucei* parasites in the blood of treated mice (data not shown). Similar results were observed in one independent experiment following intraperitoneal infection with *T*. *brucei*.

Together, these results highly suggest the correlation between the presence of the parasite, the absence of CIA development and the lower titers of anti-CII specific IgG Abs.

## Discussion

Numerous pathogenic infections can potentially impact the outcome of ADs [18]. Until recently, the majority of them were shown to promote autoimmunity, whereas a minority of infections demonstrated a protective function. In this study, we demonstrated that infection of DBA/1 prone mice with African trypanosome parasites substantially delayed the onset of a B cell-mediated autoimmune disease, namely CIA, and, therefore also plays a beneficial role. This observation is in agreement with more general and recent epidemiological data that demonstrated a reversed relationship between the incidence of parasitic diseases and autoimmunity in both the developing and the industrialized world [2].

One of the most studied case scenario is the inverted correlation between the distribution of auto-immune disorders and the incidence of the helminth infection worldwide [19]. This observation has even lead researchers to the helminthic therapy concept, which is based on the deliberate infestation of a helminth or the helminth eggs in order to treat auto-immune and other inflammatory disorders. Briefly, the immune response developed against the parasitic worm infection, mainly a Th2-skewed response, could counteract the immuno-pathological reactions, usually referred as Th1-polarised response, driving autoimmune diseases. For example, the CIA model we used in this study is induced in mice or rats after immunization of CII emulsified in CFA, which is known to polarize the cellular and humoral responses towards Th1 and the production of CII-specific IgG2a and IgG2b Abs. However, in this case, the *T*. *brucei* parasitic infection also drives a Th1-mediated response in mice [20]. Therefore the substantial impact of *T*. *brucei* infection on the onset of CIA cannot be attributed to the occurrence of a counter-neutralizing Th immune response as it is the case in helminth infection. However, in C57BL/6 mice, the “early” presence of interferon-gamma protects against the development of CIA through the suppression of Interleukin(IL)-17 [21]. Previous results by our group have put forward the impact of different African trypanosome strains on various immature and mature B cells subsets [14, 17]. In addition, we demonstrated that a *T*. *brucei* infection was able to abolish the memory or ongoing responses against unrelated antigens, such as the acellular pertussis vaccines [14, 22]. The major detrimental actors in the CIA model are the arthritogenic anti-CII specific Abs. Interestingly, we observed that the absence of RA symptoms positively correlates with a drastic decrease of CII specific Ab titers of the IgG2a and IgG2b isotypes, the main isotypes implicated in the disease [11]. In 2000, a study by Mattsson et al. showed that rats infected with *T*. *brucei* on the same day they were vaccinated with CII antigen in adjuvant, significantly exhibited a delayed onset associated with decreased titers of anti-CII IgG but without affecting T cell-mediated DTH response to CII [23]. However, postponing the infection by only a week abolishes this onset difference. These results contrast with our observation revealing a conserved shift in the appearance of the clinical symptoms of CIA even when the mice are infected two weeks post-vaccination. As *T*. *brucei*-infected DBA/1 mice succumbed from the infection within thirty days post-infection, we were not able to assess the evolution of the clinical scores beyond forty-five days post-vaccination. However, using a trypanocidal drug Berenil, we could follow the evolution of the clinical symptoms. Interestingly, we found that Berenil treatment restored the clinical signs of RA in mice that were previously vaccinated and infected. This re-emergence correlated with an increase of CII specific Ab titers of the IgG2a and IgG2b isotypes.

Most importantly, a more physiological infection approach using *T*. *brucei*-parasitized tsetse flies gave exactly the same phenotype characterized by a drastic delay in the onset of CIA, which is associated to a substantial impairment of anti-CII Ab levels of the different isotypes. However, Berenil treatment noticeably restores these titers. The decrease incidence of RA signs starting after day 45, which is associated to the reemergence of *T*. *brucei* parasites in the blood, suggest that Trypanosomes could alleviate the clinical outcome most likely via its impact on B cells. These observations again contrast with previous results done in rats showing only a clear improvement of CIA onset when *T*. *brucei* is administered the day of immunization with CII in complete Freund’s adjuvant. Other uninfectious procedures, e.g. the administration soluble CII within the eye’s anterior chamber of a mouse prior the immunization with CII emulsified in adjuvant, were shown to dampen, not the B cell response, but the T cell-mediated DTH response to CII via the induction of regulatory macrophages and CD8+ T cells [24, 25].

The CIA mouse model used in this study shares many clinical symptoms with RA in humans. Surprisingly and most interestingly, old epidemiological data have shown a lower incidence of RA in some Human African trypanosome-endemic countries of the African continent, e.g. Democratic Republic of Congo and Nigeria, compared to the one observed in some European countries at the same time [26]. With the recent development of molecular techniques, it will be really meaningful to confirm these results starting a new epidemiological study focusing on this particular theme. If the conclusions of these previous data are confirmed, understanding the molecular mechanisms used by the Trypanosomes to dampen B cell responses might lead to the development of new therapeutics against B cell-mediated AD as well as other diseases.
